# Co-designing a Vaping Cessation Program for Australian Young Adults: A Conceptual Model

**DOI:** 10.1093/ntr/ntae222

**Published:** 2024-09-24

**Authors:** Nicola Rahman, Bernadette Sebar, Ernesta Sofija

**Affiliations:** PhD Candidate, School of Medicine and Dentistry (Public Health), Griffith University, Gold Coast, QLD, Australia; Discipline Lead, Public Health, School of Medicine and Dentistry (Public Health), Griffith University, Gold Coast, QLD, Australia; Senior Lecturer in Public Health and Health Promotion, School of Medicine and Dentistry (Public Health), Griffith University, Gold Coast, QLD, Australia

## Abstract

**Introduction:**

Australian young adults (YA) report difficulties in quitting vaping. This study sought to understand what a vaping cessation program should look like from the perspective of current and former vapers, and professionals/experts involved in this health space, to inform the development of a conceptual model.

**Aims and Methods:**

Data collection was informed by Social Cognitive Theory (SCT) using co-design methodology to explore vaping cessation. Interactive workshops and semi-structured interviews were held online between March 2023 and January 2024, with data from participants’ narratives and written materials thematically analyzed.

**Results:**

YAs (18–24 years) identifying as current or former vapers (*n* = 15) and health professionals/experts’(*n* = 13) insights informed the model framework, incorporating three main elements based on environmental, personal, and behavioral factors shaping vaping cessation. Four design considerations were identified; the program needs to be affordable, accessible, appropriate, and adaptable. YAs expressed a strong preference to share their quitting journey with peers, endorsing a digital forum providing a hybrid framework of support.

**Conclusions:**

Vaping cessation is nuanced and complex requiring a multi-faceted approach targeted to the specific needs of the young adult population.

**Implications:**

The findings can be used to inform the development of a vaping cessation program tailored to YA in Australia and other similar contexts. YA perceived sharing the quitting journey and being inspired by the lived experience of others as critical components for successful vaping cessation. SCT is demonstrated to be a valuable behavior change framework for understanding vaping cessation and should be considered in future research on intervention development.

## Introduction

Nicotine vaping product (NVP), or vaping, use trends have increased rapidly over the last decade globally^1^ and in Australia.^2^ Young adults’ (YAs) (18- to 24-year-olds) current use is of particular concern in Australia at 21% between 2022 and 2023.^2^ This is a public health issue given the emerging evidence of the health effects of NVPs, including the risk of nicotine dependence.^3^ Evidence suggests many Australian young adult NVP users wish to quit, are often unsuccessful in their attempts and seek support in the process.^4^ Much of the available vaping cessation support is based on combustible smoking and fails to consider the nuances of vaping behaviors, such as the social exposure, ease of access, and perceived risks and benefits of vaping.^5^

With the growing evidence of the health risks of vaping and YAs reporting difficulties in quitting,^3,6^ there is a need for research to develop targeted evidence-based vaping cessation support.^5,7^ Previous vaping cessation research examined the challenges among adults using NVPs for smoking cessation and subsequent vaping cessation in the United Kingdom.^8^ Participants struggled with the behavioral similarity of NVPs to tobacco smoking and expressed difficulty in reducing the nicotine dose or NVP use, for fear of returning to smoking.^8^ Research on vaping cessation interventions more specifically is predominantly based in the United States. This includes a synthesis of the evidence on support modality preferences of YAs, which highlighted the low uptake of quitlines and promising results of mobile-phone-based interventions for smoking cessation.^5^ However, the efficacy of such modalities in vaping cessation remains largely unknown.^5^ A pilot study of a small U.S. sample of YAs using a telehealth intervention with contingency management illustrated the benefit of increased access to support for remote populations.^9^ Text-message intervention research includes the design of a program for Latino YAs^10^ and testing the efficacy of “This is Quitting,” a program for U.S. YAs demonstrating promising results in e-cigarette and dual e-cigarette/combustibles use.^11,12^

Recommendations for further research include the need for more global studies in differing regulatory and cultural contexts.^7^ Australia’s vaping regulation currently requires a prescription for access to either NVPs or zero-nicotine vapes and bans the sale of any vape in general retail settings.^13^ A pending change will see therapeutic NVPs sold in Pharmacies from October 2024 without the need for a prescription.^13^ Whilst the full impacts of this change are yet to be revealed, this may present as a missed opportunity for YAs visiting a General Practitioner (GP) to discuss vaping cessation support. There is a need for cessation research to develop support that considers the nuances of YA vaping.^5^ More specifically, there is a need for research that considers the lived experience of current and former vapers, to develop support targeted to their needs. To the authors’ knowledge, this is the first study to co-design a vaping cessation program in Australia’s rapidly changing regulatory environment. Co-design methodologies facilitate collaboration between the researcher and end-users, with benefits including increased confidence and satisfaction for end-users and agreement between the researcher’s aims and end-user’s needs.^14^ The theoretical framework of Social Cognitive Theory (SCT)^15^ informed this research; its personal, environmental, and behavioral constructs are considered to align closely to vaping behaviours^5^ and demonstrated to be important in the quitting process.^4^ The aims of this study are:

1. To understand what a vaping cessation program should look like—from the perspective of a vaper wishing to quit.
2. To gain insights from former vapers on their quitting experiences.
3. To gain insights from health professionals/experts currently working in this space.
4. To develop a conceptual model of a vaping cessation program.

## Methods

### Study Design and Sample Selection

This study adopted Trischler et al.’s 7-step co-design framework^,16^ engaging the researcher and end-users in an iterative process of co-design (Supplementary Material 1). Steps included resourcing information, planning the data collection processes, recruiting participants, warm-up activities, generating ideas for vaping cessation support, reflecting on the suggestions, and realizing the model. We chose to use an abductive approach to the co-design process. While deductive approaches consider the available evidence and expert knowledge, and inductive approaches the insider’s experience, an abductive approach uses a combination of the two.^17^ SCT constructs such as knowledge and social support were embedded within data collection activities, and the theoretical framework was used to facilitate our exploration and understanding of relevant phenomena.^15^ Recruitment of participants was based on the following eligibility for end-users:

1. Young adults (YA) aged between 18 and 24 years, currently living in Australia as a Permanent Resident or Australian Citizen and were a current or former vaper.
2. A health professional/expert (HP) involved in supporting young people’s health and/or in supporting young people to quit vaping.

YAs were allowed to self-identify as current or former vapers, to facilitate inclusion of the perceptions and insights of this population. YAs were recruited between March and May 2023, via an expression of interest link following completion of an earlier phase of a study reported elsewhere.^4^ This recruitment was via social media platforms such as Facebook and Instagram and flyers placed in community locations such as gyms or cafes. Further recruitment in October 2023 via the university broadcast for volunteers generated a few more YA participants. HPs were recruited between November 2023 to January 2024 via the research team’s university broadcast for research volunteers, professional networks, and LinkedIn.

### Procedure

Two YA co-design workshops of 2 hours duration were held during July 2023 online via Microsoft Teams, according to the co-design protocol (Supplementary Material 2). Online delivery accommodated participant**s’** diverse geographical locations within Australia. Additionally, three participants preferred a one-to-one session, therefore activities were adapted to a semi-structured interview based on the co-design protocol, held in October 2023 (Supplementary Material 3). Participants were provided secure access to a Padlet digital notice board, with visual prompts presenting the questions and activities. Participants could respond verbally with discussion audio-recorded, or with written notes on the Padlet.

YAs were presented with ten ideas cards, each consisting of an image and a short description of existing vaping cessation strategies based on historical smoking cessation^18^ and limited vaping cessation evidence.^19,20^ These included cold turkey, nicotine replacement therapy, weaning off vapes over a time period, calling a quitting helpline, visiting a GP, mobile phone app, YouTube videos, self-help books, support groups, and hypnotherapy. Participants provided feedback in the form of “likes/dislikes/improvements/better idea” and ranked existing strategies in order of preference from most to least preferred. YAs were then asked to generate ideas of what their ideal vaping cessation support program would look like. Questions to consider included “what types of tools or type of support do you think you need?,” “what would motivate or encourage you to engage in quitting?,” “what would help you overcome the barriers to quitting?” and “what type of communications would you like to receive and how?.”

HP workshops followed according to the same protocols, hosted in group and individual formats due to difficulties in coordinating a time suitable for all. Insights generated from the YA sessions were shared with HPs via a PowerPoint presentation, to discuss the feasibility and realization of the suggestions.

All participants were offered a $50AUD gift voucher following completion of their session, as a token of gratitude for their contributions.

### Data Analysis

Data were analyzed from the YA ideas cards feedback, preference ranks, concept generation, and HP insights. First, points were awarded according to preference ranks, with ten points allocated to the first preference, nine points to the second, and so forth. The idea with the most points reflected the participants’ overall preference. Padlet written comments and audio recordings were transcribed verbatim and thematically analyzed using NVivo software and reflexive thematic analysis.^21^ This six-step approach to qualitative analysis acknowledges the researcher cannot be fully independent of the data. Rather, the researcher brings their skills, knowledge, and experience to the process, reflecting on how this may shape their interpretation of the data.^21^ The first researcher (NR) familiarized herself with the data and identified codes in an organic and unstructured process. Codes were grouped together to generate initial themes of related meaning and these themes were iteratively reviewed and developed (NR, BS, and ES). The themes were refined, named and the final themes reported in this paper when the team was satisfied the themes were a coherent interpretation of the data.

## Results

### Participant Characteristics

Participant characteristics of YAs (*n* = 15) and HPs (*n* = 13) are provided in Table 1. Vaping status was recorded via an anonymous poll for YA WPs, to ensure their participation in a mixed group was not influenced by feelings of discomfort with this initial disclosure. The average age of YAs was 20.75 years (range 18–24). HP participant disciplines included GP, Health and Well-being Counselor, Mental health Educator, Pharmacist, Nurse, Addiction Psychiatrist, Drug and Alcohol Educator, Health Promotion Officer, Policy Advisor, and Psychologist.

**Table 1.** Participant Characteristics

Young adults			
Participant gender	Location (state in Australia)	Age	Vaping status
**Workshop 1**			
WP1	Queensland	24	4 Current vapers 2 former vapers
WP2	Queensland	22	
WP3	Queensland	19	
WP4	Queensland	23	
WP5	Victoria	19	
WP6	Queensland	20	
**Workshop 2**			
WP7	Victoria	22	3 Current vapers 3 Former vapers
WP8	New South Wales	19	
WP9	Victoria	23	
WP10	Queensland	19	
WP11	Queensland	20	
WP12	Victoria	19	
**Semi-structured Interviews**			
IP1 Female	Queensland	20	Former vaper
IP2 Female	Queensland	22	Former vaper
IP3 Female	Queensland	24	Former vaper
**Health professionals**			
**Discipline**	**Practice type**	**Location**	**Allocated code name**
General Practitioner	Community Health Center	Regional South Australia	GP 1
General Practitioner	Community Health Center	Regional Queensland	GP 2
General Practitioner	Community Health Center	Brisbane Queensland	GP 3
Health and Well-Being Counselor	Community Health Center	Gold Coast Queensland	Mental Health Counselor
Mental Health Educator	Statewide Program	Gold Coast Queensland	Mental Health Educator
Pharmacist	Community Health Center	Melbourne Victoria	Pharmacist
Clinic Nurse	Community Health Center	Gold Coast Queensland	Nurse
Addiction Psychiatrist	Drug & Alcohol Use Facility & Consultation	Brisbane Queensland	Addiction Psychiatrist
Drug and Alcohol Educator	National Audience	Sydney New South Wales	Drug and Alcohol Educator
Health Promotion Officer	Community Health Center	Regional Victoria	Health Promotion Officer
Psychologist	Community—Consultancy	Gold Coast Queensland	Psychologist 1
Psychologist	Private Practice	Melbourne Victoria	Psychologist 2
Policy Advisor	Consultancy	Australia	Policy Advisor

### Synthesis of Findings

**Figure 1.**
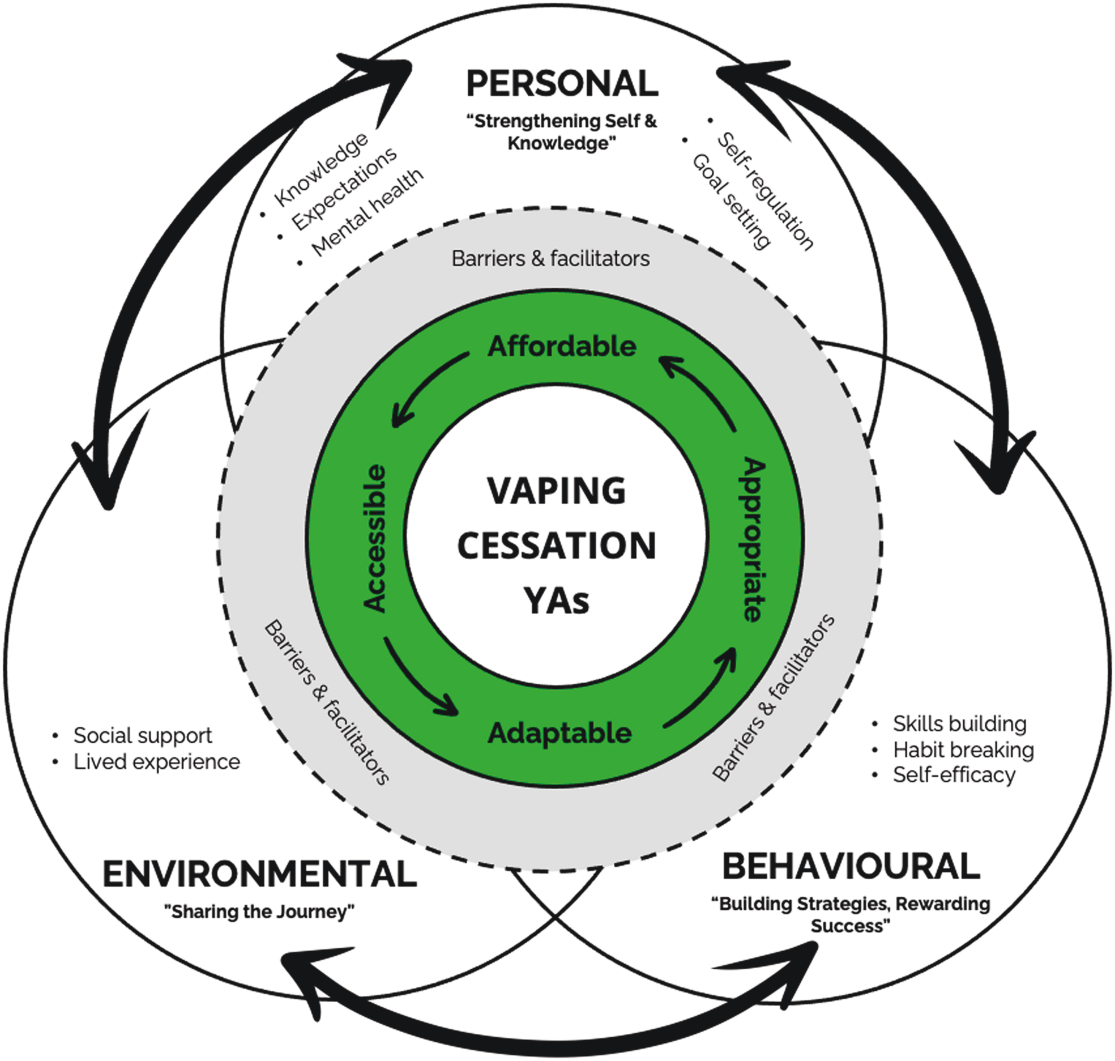
Conceptual model of a vaping cessation program.

The conceptual model (Figure 1) is informed by our research findings and based on the theoretical framework of SCT.^15^ There are three integrated elements underpinning the model relating to environmental, personal, and behavioral factors shaping vaping cessation. Within each element are components to include based on YA experiences when trying to quit and insights into HPs. Barriers and facilitators of cessation are integrated within the themes and illustrated in the model as a second revolving circle. The permeable circle wall represents how barriers and facilitators can infiltrate each element of vaping cessation. Individual ranks for vaping cessation ideas (Table 2) demonstrated a clear preference for a program to include or be based on peer group support, with strategies to address nicotine addiction and underlying mental health issues. It was evident from the data that “no one size fits all” and program delivery should be hybrid to best meet end-users’ needs, as will now be discussed. Example quotations are provided throughout the text and in Table 2, as either workshop participant (WP) or interview participant (IP) for YA, or health professional code name (as per Table 1).

**Table 2.** Cessation Strategy Preference Ranking, Participant Quote, and Design Consideration

Magnitude of preference for existing idea for vaping cessation		Participant quote - workshop participant (WP) interview participant (IP)	Social Cognitive Theory factor—conceptual model design consideration
1	Support Group	“This could definitely bring some accountability. Some people don’t have friends/family that they feel comfortable confiding with.” (WP)	*Environment—*accessibility, digital medium
		“It’s good to have a community reaching the same goal, it can be a very supportive environment.” (WP)	
2	Cold Turkey	“I like how it’s a very firm start. I don’t like how you need to have a lot of willpower. I think it can be improved if you have the right support from people around you, so you aren’t tempted.” (WP)	*Personal* – self-regulation *Environment –* support *Behavioral*—habits, addiction
		“I understand that a lot of people are a lot more addicted, and for those people it just wouldn’t work.” (IP)	
3	Weaning	“It depends a lot on self-discipline, and I personally feel that if you have the discipline to start monitoring when you do it and decrease the number of times you vape in a day, then you may as well go the extra step and stop cold turkey...” (WP)	*Behavioral—*habits, addiction *Personal –* self-regulation *Environment—*support
		“I really like this idea since it’s gentle and doesn’t force you to quit immediately, which means you have time to get used to not vaping and to get rid of the habit. It might have some drawbacks though since the continued action of vaping might just make it harder to quit.” (WP)	
4	NRT	“It just kind of reminds me of like old people like trying to get off cigarettes... also like I don’t know who can afford these things, how expensive everything is at the moment, it’s just not something you’d put on the list of things to buy.” (IP)	*Environment*—cost, support guidelines *Behavioral—*habit
		“I think if actually people had a guideline of maybe how to quit and tools to use and like when to use, how to recognize what stage they’re at, it would probably be a lot more helpful because people are quite lost in the quitting process.” (IP)	
5	Helpline	“I like it because they can offer real and helpful advice on quitting, however it might be challenging for some people since they face social criticism. Also just calling wouldn’t really help with quitting, however it would be a good place to start. Could be improved if combined with other methods.” (WP)	*Environment*—support, availability
		“I actually don’t think this would be particularly effective. I can’t imagine you’d quit, especially after being like a prevalent vaper or something, after just one phone call... and then hotlines also have a really bad name for being really busy and not picking up.” (IP)	
6	Mobile app	“... A lot of the time it does check in, ‘what’s your thoughts?’ ‘How have you felt today?... I’m also a visual learner and so visually being able to track my progress is really good. So I think for visual people it’s a really good thing.” (IP)	*Environment—*support *Personal* – goal setting
		“Yeah, just if it was like branded and coloured well... and it was easy to use... If it was a good user experience, then like I think there could be like a positive with that.” (IP)	
7	GP	“I wouldn’t do it personally. Maybe people who are like close with their GP and have an established relationship with them, but I don’t have an established relationship with my GP... especially in this age where you’re chasing a bulk billed doctor.” (IP)	*Environment*—cost, accessibility, education of health professionals
		“Most doctors aren’t knowledgeable about vaping, so going to a doctor wouldn’t really provide any insight, especially when you can call a helpline where someone with actual advice can help you.” (WP)	
8	YouTube	“Maybe like I would say TikTok’s a platform for that. I guess there could be a positive in it, but like I mean again YouTube like you by the time you click on to it find something, go through the ads, it’s just too long like and it would need to be short.” (IP)	*Environment—*support, digital medium, time limited
		“I would get bored. I’ve become addicted to TikTok... I would struggle to get through a 30-minute video.” (IP)	
9	Self-help books	“I don’t think self-help books would be so popular amongst people my age. It just seems long winded if it’s been put into a book...” (IP)	*Environment—*support, appropriate
		“It’s not popular, but maybe for older vapers.” (WP)	
10	Hypnotherapy	“I feel like this may also be very dependent on the person and I personally don’t believe in hypnotherapy.” (WP)	*Environment—*cost *Behavioral—*addiction
		“Probably most costly so if you have the funds yes, as well as if you were very addicted.” (WP)	

### Vaping Cessation Program Elements

#### Environmental—“Sharing the Journey”

The theme of YAs needing to share their quitting journey is an element incorporated into the conceptual model. The idea of a support group was expressly favored as a strategy to facilitate vaping cessation, with several WPs citing it to be *“*the best idea so far!,” “since you have that social support, as well as a sense of responsibility to see it through*.” The benefit of a support group was described as “*good for a lot of people to share their difficulties and feel heard*”* (WP) with *“...* comfort in knowing that other people feel the same way*”* (IP). One IP described shared knowledge as central to this strategy, *“*that’s where the foundation of the support group starts*”* and cited the possibility of further support emerging from this approach:

*“...sharing knowledge and sharing stories and that human connection kind of thing... once you finish the session ‘oh, I know this good GP and they like specialise in this kind of thing. Or they specialise in mental health’... here’s a number...”.*

This form of support would provide a community to reach out to, especially helpful for those who may not feel they can approach friends or family as expressed by one WP; ‘*Some people don’t have friends/family that they feel comfortable confiding with’.* The support group could cater to individuals at differing stages of their quitting journey;

*“That type of support group would definitely help because I mean, that’s what those sorts of types of meetings are for those addicted, but also for people who are doing it like just following a social trend, if they’re in a group of other people who are trying to quit as well, then they would follow that trend”* (IP).

Several participants expressed how the lived experience of others was helpful, previously observed through digital channels such as YouTube; *“*Watching people speak about it and show first-hand what they’re going thru and how they deal with it is really motivating*”* (WP). Inclusion of the lived experience of others into all modes of support delivery would contribute to the concept of sharing the quitting journey, expressed by the Mental Health Educator, *“*That whole idea of... getting people that have been through it and hearing their stories.*”*

While HPs felt the idea of shared support was an effective strategy, they voiced concerns about participant commitment if in-person attendance was required; *“*Are they actually craving connection, but in practice... Is it something that they keep up with?*”* (Addiction Psychiatrist). This could be overcome with an online delivery modality, considered suitable by several YAs; *“*I’ve heard some people succeed with just like bonding with strangers on the internet over quitting*”* (IP). While several HPs felt in-person attendance would likely be more effective, they did concur that *“*the target audience would be mostly online*”* (Nurse).

In addition to sharing their quitting journey, several YAs expressed ideas on how to address the challenge of social exposure to vaping by changing their social environment, such as *“*avoiding friends that would make me go back into vaping*”* (WP). Social support that facilitated cessation, such as “leaning on a support system*”* (WP), was also endorsed.

#### Personal—Strengthening Self and Knowledge

The personal element of the vaping cessation program addresses the knowledge of the health risks of vaping, the benefits of quitting, goal setting, self-regulation, and supporting underlying mental ill health. Participants expressed the importance of the provision of up-to-date information about the risks of vaping and the need for clearer health messaging, preferencing shock tactics and hard facts:

*“I have seen this video of someone having their vape explode in their face, as well aslots of‘scary’ infographics about the dangers. Maybe something like those drunkdriving adverts, where it tries to get people to really know the consequences” (WP).*

The use of such threat appeal^22^ was endorsed by several study participants, “You know how with smokes, we have that... everyone in your family will die if you continue to smoke... it should be the same*”* (WP). Education was considered *“*a big part of it*”* by some YAs with *“*misinformation*”* described as a contributing factor to continued vaping: *“...*because we don’t know what’s happening for sure... why is vaping actually bad? we know that it’s bad but why is it bad?*”* (WP). HPs concurred that materials should inform YAs of the known health risks of vaping such as *“*a one-page guideline mentioning the common harms of vaping because it has got nicotine added... all the side effects nicotine can cause...*”* (GP 1).

Incorporating the potential benefits of vaping cessation was also recommended, with several YAs describing their motivation to quit as the effect of vaping on their appearance, as seen on social media: *“*I’ve seen people who go, in the comments, my skin looked ashen when I was vaping, I looked lifeless. Here are the photos of me then and here are the photos of me now...!*”* (IP). The Mental Health Counselor concurred that health messaging for this population centers around fitness and body image, and promoting the attraction of vaping cessation could appeal, *“*people sharing their journeys towards getting to their health goals*.” Goal setting was a mechanism most YAs also favored to support their quitting and strengthen their self-regulation, describing existing strategies such as weaning requiring too much self-discipline. The use of a mobile app for tracking progress of goals was considered helpful and motivating: “*you get a sense of fulfillment when every day you’ve seen your tally go up and oh haven’t done that for this many days*”* (IP).

Finally, the need to support underlying mental health issues was evident. Existing strategies such as calling Quitline were considered *“*daunting*”* and potentially unsuitable by a few YAs as many of their generation had *“*phone anxiety.” *One WP expressed how “*a general mental health hotline would seem more suitable and less gimmicky,*” illustrating health needs beyond the immediate desire to quit vaping. This health need can be addressed by the integrated elements of the program. For example, strategies that facilitate vaping cessation such as mindfulness techniques and breathing regulation are also known to be useful in stress reduction and improving mental health.*^23^ Building supportive environments and the provision of social support can facilitate the recovery from mental ill health,^24^ a strategy endorsed by several YAs for vaping cessation support. This integrative support is illustrated in the conceptual model by reciprocating arrows between elements and overlapping element circles.

#### Behavioral—Building Strategies, Rewarding Success

The behavioral element of the vaping cessation program is based on the need to build skills to facilitate cessation, break the habit, and reward success. These in turn may develop self-efficacy, the individual’s confidence in their ability to quit vaping. First, planning the change was considered important by a few YAs to *“*have time to identify some alternative coping skills*”* (WP). Strategies to facilitate vaping cessation were suggested by several participants, including finding an alternative distraction such as physical activity, described as *“*a reliable way to crush a craving*”* (WP). Skills building also focused on relaxation and breathing techniques as *“*an anxiety releasing thing*,” with one WP recommending the “*necklace whistle*”* to regulate breathing and reduce anxiety. Relaxation techniques were also used by Psychologist 1 to build skills:

*“One of the really early things we often do with patients or clients is teach them some kind of relaxation technique.... just to be able to kind of centre themselves or ground themselves without reaching for the vape straight away...”*

YAs identified that strategies were required to break the habit of vaping, a limitation of existing cessation strategies: *“*What’s going to be hard is actually stopping the habit of bringing the vape to your mouth*”* (WP). Several YAs described *“*finding another habit to break that habit*”* with suggestions including *“.*.. buy gum, hard candies, toothpicks and other things you can use to help fight the urge to vape*”* (WP). The necklace whistle was again suggested as beneficial as, *“*you don’t feel like as fidgety; you have something to do with your hands*”* (WP).

One IP felt *“*there needs to be more incentives to support young people in quitting,*”* reflecting a need for feedback on the quitting journey. Existing strategies such as *“*going cold turkey*”* were described by some as most *“*consistent for relapse*”* (WP) and requiring self-discipline, but *“*could be improved with maybe a reward system*”* (WP).

#### Four As—Affordable, Accessible, Appropriate, and Adaptable

There are four main program design considerations (4As) driven by the findings and identified through the thematic analysis as central to program development; affordable, accessible, appropriate, and adaptable. These are illustrated as a focal revolving feature (Figure 1). Almost all YAs reported several of the existing cessation strategies to be costly and prohibitive as support. This was particularly evident when discussing the idea of visiting a GP with most participants unable to access a bulk-billing doctor; “…most GPs are no longer free and often cost $40-$60 for 15 minutes*”* (WP). **Affordability** was also a factor considered with other existing strategies such as nicotine replacement therapy, *“*I used to work at a chemist and know that these products can be rather expensive*”* (WP) and mobile-phone apps, *“*would be great if free!*”* (WP). HP insights concurred that cessation support must be affordable, specifically citing therapeutic support. Current nicotine replacement therapy for example is neither approved for vaping cessation purposes nor subsidized by the Pharmaceutical Benefits Scheme^25^; “… if someone gets a script from the GP and then they go to the pharmacy… it’s not Pharmaceutical Benefits Scheme subsidized, they have to pay more if they go to their GP…*”* (GP 1).

Vaping cessation support must also be **accessible**, with consideration given to the modality of support to ensure reach to all populations, including those with diverse physical and intellectual needs. Accessibility across geographical locations is also important for Australian YAs given the diverse spread of the population within and between states and territories. Some participants acknowledged a restriction of their highest ranked activity, the support group, which could be a lack of access if delivered in person; *“*Might be hard to find one*”* (WP). A solution may therefore be to deliver online support groups, an idea that was endorsed by a few YAs; *“*I think online forums... I’ve heard a lot of people my age are into it...” (IP) and ‘You need a little like group chat or something*” (WP).* Accessibility was also necessary according to one IP who felt *“everything’s so hard”* when it comes to *“trying to get something done,” highlighting the importance of making engagement easy.*

Next, delivery modality and resources must be **appropriate,** targeted to the YA population. The use of suitable language and preferred digital platforms are just a few examples cited by almost all participants: *“*Platforms that young people use the most, it would help that there be vape cessation information e.g.: TikTok info videos rather than ABC radio...*”* (WP). This was confirmed by HPs when discussing the possible use of a mobile app: *“*...it would have to pass their kind of sniff test... to make sure it didn’t seem like dorky or adults trying to use their lingo*”* (Addiction Psychiatrist). The program must consider how YA live their lives when addressing their needs, to attract YAs thinking about quitting vaping and maximize the efficacy of their quit attempts. This was reflected in a discussion with HPs: *“...*people doing it in combination with their friends, using an app on the phone that they do everything else in their life with, with the support group online of other people is how we might make progress*”* (Policy Advisor).

Finally, the program must be **adaptable**, facilitating a flexible approach for YAs to choose preferred elements of support. End-users may seek differing kinds of help or be at varying stages of the quitting process and therefore program flexibility to suit the individual will ensure there is something for everyone. A hybrid approach is suggested to address this design consideration. This idea was positively endorsed by several HPs such as GP 2, *“*There’s gotta be multiple, multimodal sort of strategies for it*”* and the Addiction Psychiatrist: *“*Somewhere...where they had kind of a place of options, and they could take what they wanted... a scaffold to help with reducing [vaping]... where they could pick and choose what worked for them*.” This approach would also support the varying ways people learn and retain information:*

*“... so you’ve got people who will sit and listen to a talking head, because they like that auditory kind of approach... maybe in a support group situation just talking is enough, but you’ll have some people who need something a bit more visual than that... that’s where your app comes in...”* (Psychologist 1).

Adaptability is also essential to ensure the program is sustainable and consistent with ongoing needs, given the nature of Australia’s rapidly changing regulatory environment.^13^ This design consideration will ensure support systems incorporate the most recent evidence-based rationale.

## Discussion

This is the first study to co-design a vaping cessation program for young adults (YAs) in the regulatory context of Australia; addressing the call for vaping cessation research in differing regulatory and cultural contexts.^7^ Co-design workshops with YAs and health professionals/experts (HPs), the theoretical framework of SCT,^15^ and our previous findings^4,26^ informed the development of a conceptual model for vaping cessation. The model illustrates the integrated vaping cessation support elements; environmental, personal, and behavioral. Within each element are program components and design considerations that address the specific needs of YAs when considering quitting vaping. Our empirical findings advance our understanding of the nuances of YA vaping cessation and the suitability of SCT as a framework to enhance vaping cessation programs. Specifically, our findings highlight key considerations.

Our findings suggest that vaping cessation is multi-factorial and requires a suitably comprehensive and targeted approach.^6^ Future vaping cessation support for YAs should be comprised of the strong preference of this age group to “share their quitting journey” with peers and be inspired by the lived experience of others. Analysis of the data demonstrated this environmental component should be foundational in the program design. Environmental factors such as social support are not addressed in many current cessation strategies, such as Quitline’s support based on counseling and access to pharmacotherapy.^27^ Quitline has demonstrated success in smoking cessation in Australia^28^ and in vaping cessation overseas,^29^ however its success for Australian YAs vaping cessation is not evidenced. The provision of a hybrid framework of support incorporating multiple components such as social support, building skills for cessation, strengthening self-regulation, and increasing knowledge of the risks of vaping is required. The provision of such a targeted approach increases end-user satisfaction and leads to improved health outcomes.^14^

Digital delivery of this targeted model would ensure the program design is affordable, accessible, appropriate, and adaptable. There is growing evidence of the cost-effectiveness of digital health interventions^30^ and their potential to maximize reach to marginalized populations, both geographically and those with special or minority needs.^31^ Digital modalities have demonstrated success in vaping cessation in overseas populations, such as text-message interventions,^7,11,12^ mobile apps,^32^ and internet-based support.^33^ However, there is a need for digital vaping cessation support that incorporates behavior change components^34^ and is targeted to YA.^33^ For example, reward has been demonstrated to be an important factor in health behavior change theory,^35^ and suggested by participants as a strategy for facilitating cessation. Providing information on the tangible rewards of money saved or the health benefits of quitting serves to reinforce changed behavior and strengthen self-efficacy, as illustrated in the behavioral element of the model. Another successful behavior change mechanism is the use of threat appeal to provide health risk information,^22^ also proposed by YAs in this study. Both YA and HP participants in this study endorsed a digital forum that integrates all factors shaping vaping cessation; a one-stop shop from which YAs can draw according to their needs. The conceptual model addresses the design considerations to incorporate delivery of support appropriate for YAs, whilst informed by the behavior change framework of SCT.^15^

### Strengths and Limitations

The key strength of this study is the exploration of the lived experience of YAs when attempting to quit vaping, facilitated by the use of co-design methodology. Exploring the experiences, views, and insights of YAs who have tried to quit or have successfully quit informs our understanding of the current contextual factors shaping their vaping behaviors. There is limited research on YA experience of vaping cessation, with previous lived experience research focusing on adolescents’ e-cigarette use in overseas populations^36,37^ and Western Australia.^38^ Vaping cessation support was recently explored in school-aged youth in New South Wales, Australia.^39^ Involving YAs in the design ideation of the conceptual model ensures agreement with end-users’ needs in this age group.

Limitations to acknowledge include the challenge of recruiting young adult participants, despite incentivization and initial interest of large numbers. This may be due to the current lifestyle of this age group, with multiple demands and cost of living pressures. The sample may be open to self-selection bias given the nature of terminology used in recruitment materials sought participant interest in research on “vaping cessation.” Finally, whilst a vaping status poll was taken at the beginning of the workshops to understand the sample characteristics, this poll was anonymous to encourage participants**’** contributions free from judgment on their vaping behaviors. Differentiating between current and former vapers’ insights may be beneficial to our understanding of vaping cessation.

## Conclusion

Our study demonstrates that a successful vaping cessation program for YAs should include components that offer YAs a platform to “share their quitting journey,” provide social support, provide education on the health risks/benefits and facilitate empowerment. The exploration of the lived experience of YAs when quitting vaping and the insights of health professionals/experts working in this space facilitated our understanding of the nuances of vaping cessation. Informed by the theoretical framework of SCT, we present a conceptual model that addresses the unique needs of this YA population. The model provides a framework on which to base future vaping cessation support. This framework enables the delivery of a targeted and comprehensive program that is affordable, accessible, appropriate, and adaptable.

## Supplementary material

Supplementary material is available at *Nicotine and Tobacco Research* online.

ntae222_suppl_Supplementary_Materials_1

ntae222_suppl_Supplementary_Materials_2

ntae222_suppl_Supplementary_Materials_3

## Data Availability

Data from this qualitative study are not publicly available to minimize risk from deductive disclosure and in the interests of protecting the professional occupations of participants.
