# Supplementary material for: Co-designing a Vaping Cessation Program for Australian Young Adults: A Conceptual Model
Source: Nicotine Tob Res. 2024 Sep 24;27(3):457–65. doi: 10.1093/ntr/ntae222 (PMC11847777; doi:10.1093/ntr/ntae222)
Supplement: ntae222_suppl_Supplementary_Materials_1 [file ntae222_suppl_supplementary_materials_1.docx]

**Supplementary Material 1**

*Overview of the seven-step co-design framework*

| **Co-design stage** | **Application in practice and considerations** |
| --- | --- |
| *Steps 1-3 occurred prior to the co-design workshops.* | |
| 1. Resourcing | Insights gained from the literature review informed the study design and the focus of activities for the workshops. Existing vaping cessation themes and quitting strategies were identified. The research team agreed on Social Cognitive Theory [SCT] constructs on which to base data collection processes. |
| 1. Planning | Regular meetings of the research team were held to organise and plan the co-design process and workshops, including data collection measures, questions and activities. This was an iterative process due to unexpected logistics in recruitment, such as difficulty recruiting young adults [YA] and accommodating diverse participant locations. |
| 1. Recruiting | Participants were recruited through a diverse range of mediums including social media platforms, community-based flyers and professional organisations. A gift voucher was offered as a token of appreciation for contribution. Recruitment was an iterative step to maximise possible participation. |
| 1. Sensitisation | Sensitisation activities occurred during workshops, as opposed to before, due to participant accessibility and time constraints of target population. |
| 1. Facilitation   *(see Supplementary Material 2 for protocol and measures)* | Three phases to the workshop:  *1) Introduction:*  Use of warm-up activities and preparation of participants for co-design.  *2) Sensitisation* activities:  - Thought provoking questions based on SCT constructs (e.g., knowledge, expectations, social support), designed to elicit knowledge and insights of participants whilst building their confidence and rapport  - Use of ‘ideas cards’ representing existing cessation strategies used (based on literature review), participants reported ‘likes/ dislikes/ improvements/ better idea’ feedback to each idea and finally, participants ranked the ideas in preferred order.  *3) Design ideation activity and pitch presentation (YAs only):*  *-* Participants discussed in groups their ideal vaping cessation support, were encouraged to develop strategies of ideas cards or generate new ideas. Participants reported back via recorded discussion and written notes. |
| *Steps 6 and 7 occurred after YA workshop completion.* | |
| 1. Reflecting | YA findings and insights were shared with HPs as part of the iterative co-design process in subsequent workshops, to gain further insight into support service provision.  All qualitative data were analysed (written notes and discussion recordings), based on Braun & Clarke’s Reflexive Thematic Analysis (2021). Code development and theme organisation was agreed by research team, whilst abductively considering the SCT constructs and theoretical framework. |
| 1. Building for change | The design-informed conceptual model is formalised. This step is iterative in nature and will form the basis of the next study to ‘realise’ the current model in partnership with designers, external organisations and service delivery experts, before piloting. |
