# Supplementary material for: Co-designing a Vaping Cessation Program for Australian Young Adults: A Conceptual Model
Source: Nicotine Tob Res. 2024 Sep 24;27(3):457–65. doi: 10.1093/ntr/ntae222 (PMC11847777; doi:10.1093/ntr/ntae222)
Supplement: ntae222_suppl_Supplementary_Materials_2 [file ntae222_suppl_supplementary_materials_2.docx]

**Griffith University GU Ref 2022/925**
**Vaping Cessation Co-Design Online Workshop – Protocol**

| **PART 1: Introductions & Background information**  **Duration: 5 minutes** | **Key Outcomes** |
| --- | --- |
| **Objectives:** Inform participants of the purpose and nature of the study; Give a general background to the research project; Advise participants of ethical requirements; Set expectations for group outcomes. | |
| **10.00 – 10.05 Begin - Confirm Consent from all participants and if not emailed give verbal consent recorded.**  *Thank you very much for agreeing to participate in today’s session.*  *My name is [Name] from [Institution], and the team members are [names introduced].*  ***Before we begin, there are a few things we need let you know:***   - Mobile phones to silent - Please use your microphone/camera when speaking - Recording   ***Before we start this session, I will explain why we are here today.***  *We are conducting this workshop today to come together to contribute to the design of a program that helps young adults quit vaping. Nicotine Vaping Product (NVP) use, otherwise known as vaping, is a critical public health issue with increasing prevalence amongst young adults in Australia. The risk of nicotine dependence is known and there is a three-fold increased risk of smoking cigarettes following vaping initiation.*  *The aim of this research is to explore the experience of vapers when thinking about quitting, when trying to quit or how they successfully quit.*  *We have asked for your participation because we want to understand how best to support those who are vaping to quit.*  *We want to know what young adults think will support them in the quitting process, what* ***tools*** *are needed, and how best to* ***deliver*** *the support.*  *The insights that you share with us today will be used to inform a conceptual model of a vaping cessation program.*  ***Before we start, we want to remind you:***   - *that your participation today is voluntary. If at any time you do feel distressed or uncomfortable in any way you are free to withdraw from the workshop at any time.* - *we want everyone to feel safe and comfortable to speak freely and share their thoughts without judgement from other participants. It is important that you respect the opinions and ideas of other participants and importantly there are no right or wrong answers.* - *today's discussions will be recorded and any names, locations and other factors that may identify people will be removed from our transcripts. We will not be sharing any personal information (name and any other identifier) when reporting on the work that we are conducting today. This information is held exclusively by the research team.* - *this workshop will run no longer than 2hrs and you will receive a voucher to the value of $50 on completion of the workshop. Only participants who complete the session are entitled to receive a voucher.*   ***Today’s workshop consists of three main parts:***   1. *First, we will ask you some questions thoughts or experiences about the topic* 2. *Next, we will ask you to provide feedback on a range of ideas that could be used to quit vaping* 3. *Then, we will ask you to offer your own ideas. You will work in a group/ two groups to generate ideas which you feel will best support a vaper in the quitting process.*   ***Do you have any questions before we begin?***  *Feel free to turn on your microphone and camera when talking, we’d love to see and hear you!* | Participants feel welcome, comfortable, informed and able to voluntarily participate in the workshop  ***Materials and tools***  *-* Co-design discussion guide  - Participant attendee list  - Information sheets  - Consent forms gathered |
| **PART 2: Sensitisation activities**  **Duration: 55 minutes** | **Key Outcomes** |
| **Objectives:**  To understand experiences of young adults when considering quitting vaping or trying to quit.  To explore the knowledge/views of health professionals on vaping cessation and share insights from young adults. |  |
| **10.05 – 10.10 Online icebreaker and practice using the Padlet which is now shared in the chat**  *A vaping status poll will be completed anonymously for young adults.*  3 warm up activities - chance to practice using the Padlet/microphone/camera and ask questions.  **10.10 – 10.30 Thought-provoking questions (Social Cognitive Theory [SCT] domains explored) – 20 mins**  Participants will be asked to answer the following thought-provoking questions:   1. What do you know about quitting vaping? *(SCT- Personal- e.g., knowledge) -* brainstorm and type onto the notes - knowledge, challenges, risks, benefits, barriers, methods. 2. Why might young people find it difficult to quit vaping? *(SCT - Behavioural, Environmental, Personal - e.g., expectations) - taking it in turn,* each participant writes an answer to the question and the next participant reads the first note and writes something different, and then the third participant writes something different again. Or alternatively discuss.   3a. Young adults - Who would you (did you) approach for support to help you quit? *(SCT - Environmental -e.g., social support) - participants type onto the notes and then as a group discuss ‘why’ - recorded.*  3b. Health Practitioners: Questions 1 and 2, and Who do you think young adult vapers approach for support to help them quit? What do you think are the current challenges to quitting interventions?  **Activity – Ideas Cards – 20 mins** – look at the ‘ideas’ cards images on Padlet that illustrate current cessation tools/methods and discuss them, and complete feedback (likes, dislikes, improvements, better idea) for each idea with written notes on Padlet and/or discussion.  The young adult participants are asked to rank them all 1 (most preferred) to 10 (least). The option of presenting their own idea(s) is also provided.   \| **Activity** \| **Image** \| **Description** \| \| --- \| --- \| --- \| \| Going Cold Turkey \| **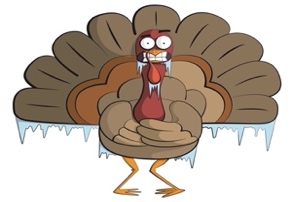** \| Deciding to quit vaping, setting a date and time to stop and just not vaping. \| \| Nicotine Replacement Therapy \| **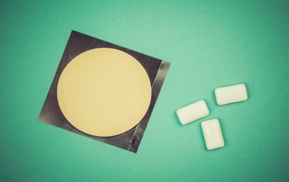** \| Using a form of replacement nicotine (e.g., gum, patches, sprays, inhalers, lozenges). \| \| Weaning off Vapes \| **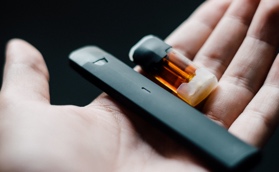** \| Weaning off nicotine and/or vapes over a period of time. \| \| Call a Helpline \| **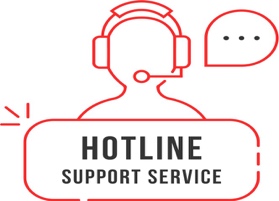** \| Call a free and confidential counselling service for advice on how to quit. \| \| Visiting your GP \| **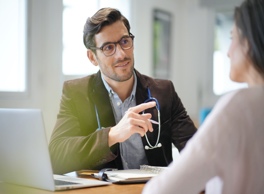** \| Make an appointment with your General Practitioner to discuss quitting vaping. \| \| Mobile phone App \| 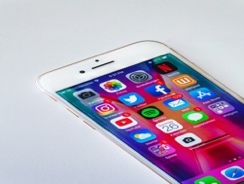 \| Downloading a ‘Quit Vaping’ app on your mobile device \| \| YouTube videos \| 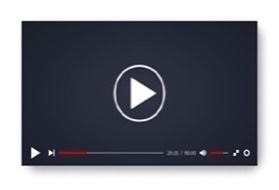 \| Watch videos on how to quit vaping \| \| Self-help books \| 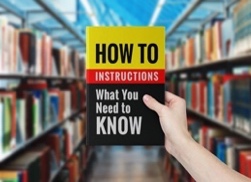 \| Read self-help material/literature on how to quit vaping \| \| Support Groups \| 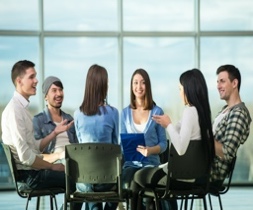 \| Join and attend a support group for help with quitting vaping \| \| Hypnotherapy \| 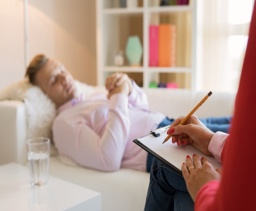 \| Using hypnotherapy via a therapist to help quit vaping \| \| Your idea? \| 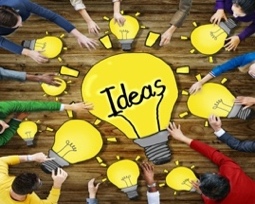 \| What other ideas do you have that you feel would better help quitting vaping? \|   **10 minute break for bathroom/ drinks** | To capture participants knowledge and thoughts on this topic, their perceptions and awareness of existing tools, and familiarise them with current solutions.  ***Materials and tools:***   - Icebreaker questions - Padlet - Activities/Ideas cards images |
| **PART 3: Co-design – Activity 5 - concept generation – Young adults**  **Duration: 30 minutes** | **Key Outcomes** |
| **Objectives:** Generate innovative user-generated ideas on appealing strategies that can be implemented in community | |
| *Introduction:*  *The rise in prevalence of vaping amongst young Australian adults is of concern. We do not understand the long term health impacts yet, we know that nicotine is highly addictive, and we know there is a three times higher risk of smoking after vaping initiation. There are limited programs and services available to help support vapers to quit, and many are based on smoking cessation. Vaping is a nuanced behaviour, and it is important to understand what current vapers need for a cessation program.*  **11.00-11.30 Design task - Idea generation**  *We want you to suggest ideas, tools and methods that would appeal to you when considering quitting vaping or through the quitting process. You are welcome to use any of the existing ideas, adapt or improve them, or ignore them altogether.*  *Please work together to design your ideal vaping cessation program.*  *Using the Padlet provided, we would like you to work in 2 teams to develop your ideas. Type your ideas on the screen. If you found any of the previous ideas effective, then mention this as part of your ideas. When using these ideas, you can modify them, so they are more appealing and engaging to you, or come up with something completely different/brand new.*  *The campaign should include the following considerations:*   - *What types of tools or type of support do you think you need?* - *What would motivate or encourage you to engage in quitting?* - *What would help you overcome the barriers to quitting?* - *What type of communications would you like to receive (for example, what type of information would you like to know about, how would you like to receive this information)?*   **[Facilitators to be available to answer questions and prompt further discussion, but to allow the participants to generate their own ideas without bias or persuasion]** | To generate end-user ideas on how best to support vapers when trying to quit, identify if there are specific options that appeal more, to understand what is most valued.  ***Materials and tools***  - Padlet  - audio-recording |
| **PART 4: Pitch presentation – Young adults**  **Duration: 15 minutes** | **Key outcomes** |
| **Objectives:** To gain an accurate understanding of each groups final design ideas; Understand which ideas are appealing | |
| **11.30** - Allow each team to present their final design ideas to the wider group and record the discussion/presentation  To present their notes/diagrams/ideas on Padlet and discussion.  RECORD/FILM PITCH PRESENTATIONS, screenshot the Padlet comments.  **Young adults concept generation is shared with health professionals in separate workshops.** | To gain an accurate understanding of each groups final design idea  ***Materials and tools***  - Audio-recording of Teams  - Screenshots |

| **PART 5: Closing**  **Duration: 5 minutes** |  |
| --- | --- |
| **Objectives:** End the session | |
| - Inform participants that it is the end of the workshop and thank them for their time and input. - Final check-in will with all participants. - State that as this is university research, it is carried out in compliance with the ethical guidelines and requirements provided in the Informed Consent Form and will only be used to inform the vaping cessation model and outcomes will be reported in research. - Remind them that you are from Public Health @ Griffith. Remind them of contact details in the Informed Consent Form and that if they would like to be kept updated with results (if so, collect their details and send them a summary report of the results once finished) - Inform them of what happens in the project from here. A reminder that the information they use will be helping to develop future vaping cessation support programs. - Ask for any final comments | To finalise the discussion and their role in this process.  ***Materials and tools***  - Gift cards will be emailed |
