# Supplementary material for: Co-designing a Vaping Cessation Program for Australian Young Adults: A Conceptual Model
Source: Nicotine Tob Res. 2024 Sep 24;27(3):457–65. doi: 10.1093/ntr/ntae222 (PMC11847777; doi:10.1093/ntr/ntae222)
Supplement: ntae222_suppl_Supplementary_Materials_3 [file ntae222_suppl_supplementary_materials_3.docx]

**Developing a co-designed vaping cessation program for young adults**

**Semi-structured Interview Protocol**

GU Ref No: 2022/ 925

**Introduction Script**

My name is [Name], I am a PhD researcher at Griffith University and this study forms part of my thesis. We are conducting this interview today to come together to contribute to the design of a program that helps young adults quit vaping. Nicotine Vaping Product (NVP) use, otherwise known as vaping, is a critical public health issue with increasing prevalence amongst young adults in Australia. The risk of nicotine dependence is known and there is a three-fold increased risk of smoking cigarettes following vaping initiation.

The aim of this research is to explore vaping cessation, the factors that may shape cessation and how best to support young people trying to quit.

We have asked for your participation because we want to understand how best to support those who are vaping to quit.

**Questions**

Before we start, you have had the opportunity to read the Study Information and Consent, but do you have any questions you would like to ask?

**Consent**

I confirm that you have provided signed consent to participate in this study, after reading the Information provided. I would like to stress that if at any time you do not wish to answer a question you do not have to do so. Likewise, if you wish to withdraw from the research, you can do so at any time prior to data de-identification. Not answering a question or withdrawing prior to de-identification will not affect the study in anyway and will not have any effect on your studies or grades if you are studying at Griffith University.

**Confidentiality**

All personal information will remain confidential, and only accessible by the research team. Your identity will be allocated a code following the interview and this code will be used to identify the interview transcription.

With your permission, I will be using audio-recorder to record the interview. This will allow for accurate capture of the information you provide. Following the interview, the audio will be transcribed and then deleted.

If you do not wish to be audio-recorded I will take notes.

Are you comfortable to now commence the interview?

Yes, continue.

No – is there anything you would like to ask? If you wish to withdraw that is completely acceptable.

**I would now like to ask a few questions about your eligibility and background:**

**Participant Information -** young adults

Name…………………………………………………………………………………….

Gender...............................................................................................................................

I would just like to confirm the following:

Are you either a current or former vaper? Yes No

Are you aged between 18-24 years? Yes No

Are you an Australian Citizen or Permanent Resident living in Australia? Yes No

**Participant Information -** Health professionals – Can you please confirm your professional role and location.

**Interview Questions:**

1. What do you know about quitting vaping?
2. Why might young people find it difficult to quit vaping?

3. Young adults - Who would you (did you) approach for support to help you quit?

1. Health professionals - Who do you think young adult vapers approach for support to help them quit? What do you think are the current challenges to quitting interventions?
2. What do you think of the following ideas for use in quitting vaping? Likes/ dislikes/ improvements/ better idea?

- Going cold turkey
- Nicotine Replacement therapy
- Weaning off vapes
- Calling a helpline
- Visiting your GP
- Mobile App
- YouTube videos
- Self-help books
- Support groups
- Hypnotherapy

1. What would your ideal vaping cessation program look like? (young adults)

Prompts can include the following:

What types of tools or type of support do you think you need?

What would motivate or encourage you to engage in quitting?

What would help you overcome the barriers to quitting?

What type of communications would you like to receive (for example, what type of information would you like to know about, how would you like to receive this information)?

**Closure**

Thank you very much for your participation in today’s study interview.

Your participation is valuable in contributing to the body of knowledge and our understanding of this health topic.
